# Culture of Human Rotaviruses in Relevant Models Shows Differences in Culture-Adapted and Nonculture-Adapted Strains

**DOI:** 10.3390/ijms242417362

**Published:** 2023-12-11

**Authors:** Nazaret Peña-Gil, Walter Randazzo, Noelia Carmona-Vicente, Cristina Santiso-Bellón, Roberto Cárcamo-Cálvo, Noemi Navarro-Lleó, Vicente Monedero, María J. Yebra, Javier Buesa, Roberto Gozalbo-Rovira, Jesús Rodríguez-Díaz

**Affiliations:** 1 Department of Microbiology, School of Medicine, University of Valencia, Av. Blasco Ibáñez 15, 46010 Valencia, Spain; nazaret.pena@uv.es (N.P.-G.); noelia.carmona@uv.es (N.C.-V.); cristina.santiso@uv.es (C.S.-B.); roberto.carcamo@uv.es (R.C.-C.); noemi.navarro@uv.es (N.N.-L.); javier.buesa@uv.es (J.B.); 2 Instituto de Investigación INCLIVA, Hospital Clínico Universitario de Valencia, 46010 Valencia, Spain; 3 Department of Preservation and Food Safety Technologies, IATA-CSIC, Av. Agustín Escardino 7, 46980 Paterna, Spain; wrandazzo@iata.csic.es; 4 Department of Biotechnology, IATA-CSIC, Av. Agustín Escardino 7, 46980 Paterna, Spain; btcmon@iata.csic.es (V.M.); yebra@iata.csic.es (M.J.Y.)

**Keywords:** human rotavirus, cell culture, glycocalyx, human intestinal enteroids, histo–blood group antigens

## Abstract

Rotavirus (RV) is the leading cause of acute gastroenteritis (AGE) in children under 5 years old worldwide, and several studies have demonstrated that histo–blood group antigens (HBGAs) play a role in its infection process. In the present study, human stool filtrates from patients diagnosed with RV diarrhea (genotyped as P[8]) were used to infect differentiated Caco-2 cells (dCaco-2) to determine whether such viral strains of clinical origin had the ability to replicate in cell cultures displaying HBGAs. The cell culture-adapted human RV Wa model strain (P[8] genotype) was used as a control. A time-course analysis of infection was conducted in dCaco-2 at 1, 24, 48, 72, and 96 h. The replication of two selected clinical isolates and Wa was further assayed in MA104, undifferentiated Caco-2 (uCaco-2), HT29, and HT29-M6 cells, as well as in monolayers of differentiated human intestinal enteroids (HIEs). The results showed that the culture-adapted Wa strain replicated more efficiently in MA104 cells than other utilized cell types. In contrast, clinical virus isolates replicated more efficiently in dCaco-2 cells and HIEs. Furthermore, through surface plasmon resonance analysis of the interaction between the RV spike protein (VP8*) and its glycan receptor (the H antigen), the V7 RV clinical isolate showed 45 times better affinity compared to VP8* from the Wa strain. These findings support the hypothesis that the differences in virus tropism between clinical virus isolates and RV Wa could be a consequence of the different HBGA contents on the surface of the cell lines employed. dCaco-2, HT29, and HT29M6 cells and HIEs display HBGAs on their surfaces, whereas MA104 and uCaco-2 cells do not. These results indicate the relevance of using non-cell culture-adapted human RV to investigate the replication of rotavirus in relevant infection models.

## 1. Introduction

According to the World Health Organization (WHO), diarrheal diseases are one of the top 10 global causes of death worldwide [1]. Moreover, in low-income countries, they rank in the top five. Among all the viral infections that cause acute gastroenteritis (AGE), rotavirus (RV) is one of the most important causes in children under 5 years of age. In addition to diarrhea, RV infection causes vomiting, dehydration, discomfort, and fever [2].

RV belongs to the *Sedoreoviridae* family, and its genome is fragmented into 11 segments. Its capsid is composed of a core layer of VP2 protein, an intermediate layer made of VP6, and an outer shell made of glycoprotein VP7 and protease-sensitive protein VP4 [3]. VP4 is post-translationally cleaved into VP8* and VP5* [4]. The first is responsible for cellular attachment and cell entry [4]. RV genotypes are defined according to the VP7 protein, which defines the G genotype, and the spike protein VP4, which defines the P genotype. Currently, G1P[8], G2P[4], G3P[8], G4P[8], G9P[8], and G12P[8] account for more than 90% of the globally circulating strains [5,6,7].

The introduction and expansion of the RV vaccine has been responsible for decreasing the mortality of children younger than 5 years due to diarrhea since 2006 [8]. Two of the available vaccines, Rotarix and Rotateq, are less effective in low-income regions such as Africa and Asia [9,10,11,12]. Among others, one of the reasons could be attributed to the diverse host mucosal factors, such as the polymorphisms of histo–blood groups antigens (HBGAs), complex carbohydrates present on the surface of gastrointestinal epithelial cells and secretions [13,14,15,16].

RV attachment to host cells is mediated by HBGAs. Their synthesis is catalyzed by FUT2 and FUT3 glycosyltransferases. Polymorphisms in these enzymes lead to the presence or absence of certain HBGAs, which affects the interaction, susceptibility, or resistance to pathogens that bind HBGAs [17,18,19,20]. Secretor-positive individuals, who express H type-1 and Lewis b (Le^b^) antigens, among others, are more common in Europe, North America, Central Asia, and some African populations. However, several African countries have a higher rate of Lewis-negative individuals, who do not express Le^a^ or Le^b^ [21].

It has been found that the RV VP8* subunit binds HBGAs in a P genotype-dependent manner [4,22,23]. Although some discrepancies have been found, studies have shown that P[8] binds to H type-1 antigen [24] and Le^b^ [16], whereas P[6] binds to H type-1 antigen only [24,25]. Rotarix and Rotateq vaccines contain the P[8] genotype [26], so they might not protect against RV P[6] infections. This could be why Lewis-negative individuals in regions where the P[6] genotype is more common continue to suffer from RV infections, as occurs in Africa.

In vitro studies on RV infectivity mainly rely on the use of cell culture-adapted strains, such as human Wa (G1P[8]) and simian SA11 (G3P[2]) strains. This is due to their capability for infection in cell cultures, in contrast to RV clinical isolates. These strains are usually propagated in the MA104 cell line, derived from monkey kidney, which is not the natural cell type for human RV infection in vivo and lacks HBGAs on its surface [27,28,29,30,31,32,33]. In addition, it has been shown that VP8* from RV Wa displays differences in binding ability to target glycans [24]. However, binding to HBGAs is an essential process in natural human RV infections [20]. Therefore, testing non-adapted human RV isolates (e.g., directly isolated from clinical samples) in cell lines that present similar surface characteristics to those of gastrointestinal cells should be preferred to comprehensively investigate human RV infectivity. In order to gain insights into these questions, RVs obtained from RV-positive stool samples, as well as cell culture-adapted Wa strain, were used to infect cell lines displaying (e.g., dCaco-2, HT29, and HT29M6 cells and HIEs) or not displaying HBGAs (e.g., uCaco-2, and MA104), to determine if there is any difference in infection depending on the virus or cell line used.

## 2. Results

### 2.1. RV from Clinical Samples Infect Diferentiated Caco-2 Cells

**Figure 1:**
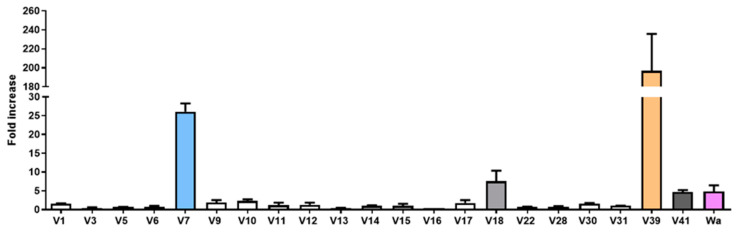
Viral replication of 21 RV-positive stool samples that attached to dCaco-2 and the culture-adapted RV strain Wa in dCaco-2 cells at 72 hpi. dCaco-2, differentiated Caco-2; hpi, hours post-infection.

Human RV stool filtrates were prepared as described in the Materials and Methods section of this article and were employed to infect differentiated Caco-2 (dCaco-2) cells and a human enterocyte cell line for a 72 h period. In order to assess viral replication, viral RNA was quantified at 1 h post infection (hpi), representing viral adhesion to the cell culture, and at 72 hpi, representing the peak of infection. The results showed that 4 (V7, V18, V39, and V41) of 41 RV clinical samples efficiently replicated in dCaco-2 cells (Figure 1). The cell culture-adapted RV Wa strain (G1P[8] genotype) showed limited replication, as observed for V18 and clinical samples. The remaining samples not represented in Figure 1 (e.g., V19, V20, or V29) were unable to attach to cultured cells (i.e., no RV detection at 1 hpi or 72 hpi). Among other factors, including the viral strain, this limited in vitro infectivity of RV-positive samples could be caused by the presence of contaminants in the stools, which prevented infection in the cell culture [20].

The V7 and V39 RV clinical samples were selected for further analysis. Their VP4 and VP7 encoding genes were sequenced, and both displayed the G9P[8] genotype. The nucleotide and aminoacidic sequences of both strains were identical in the case of VP4 (Supplementary Figure S1). Regarding VP7, there were two nucleotide variations in the nucleotide sequences of V7 and V39, whereas aminoacidic sequences were identical (Supplementary Figure S2).

**Figure 2:**
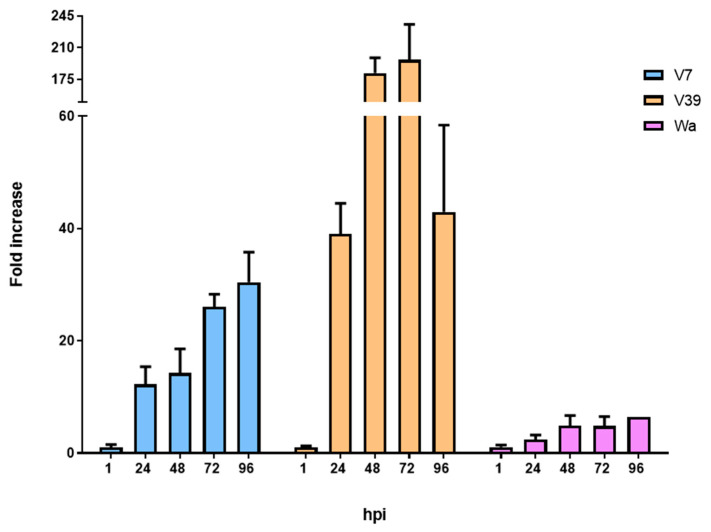
Time-course infection of stool filtrates V7 and V39 and the culture-adapted RV Wa strain at 1, 24, 48, 72, and 96 hpi in dCaco-2 (differentiated Caco-2 cells).

The selected viral filtrates, along with RV Wa, were then used to perform a time-course infection in dCaco-2 cells for 1, 24, 48, 72, and 96 hpi (Figure 2). The replication of all viruses sequentially increased up to 72 hpi. However, at 96 hpi, the viral titers decreased compared to 72 hpi for V39. In light of these results, a standardized infection endpoint of 72 hpi was chosen in subsequent experiments for all viruses.

**Figure 3:**
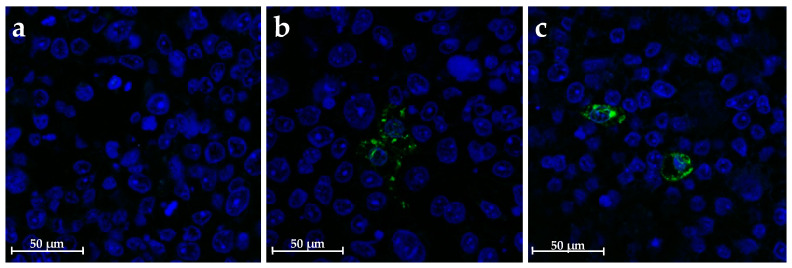
Immunofluorescence of dCaco-2 cells infected with the virus filtrates at 24 hpi. (**a**) mock infected cells, (**b**) cells infected with V7 filtrate and (**c**) cells infected with V39 filtrate.VP6 signal is shown in green, and nuclei were stained with DAPI (blue). Magnification 40×. dCaco-2, differentiated Caco-2; hpi, hours post infection.

To further demonstrate the replication of RV from stool filtrates in dCaco-2 cells, immunofluorescence detection of infected cells was performed at 24 hpi using isolate V7. Figure 3 shows the infected dCaco-2 monolayer with DAPI-stained nuclei (blue), in which some cells display a VP6 signal in their cytoplasm (green), indicating RV replication.

### 2.2. Viral Replication in Relevant Cell Culture Models Expressing HBGAs

**Figure 4:**
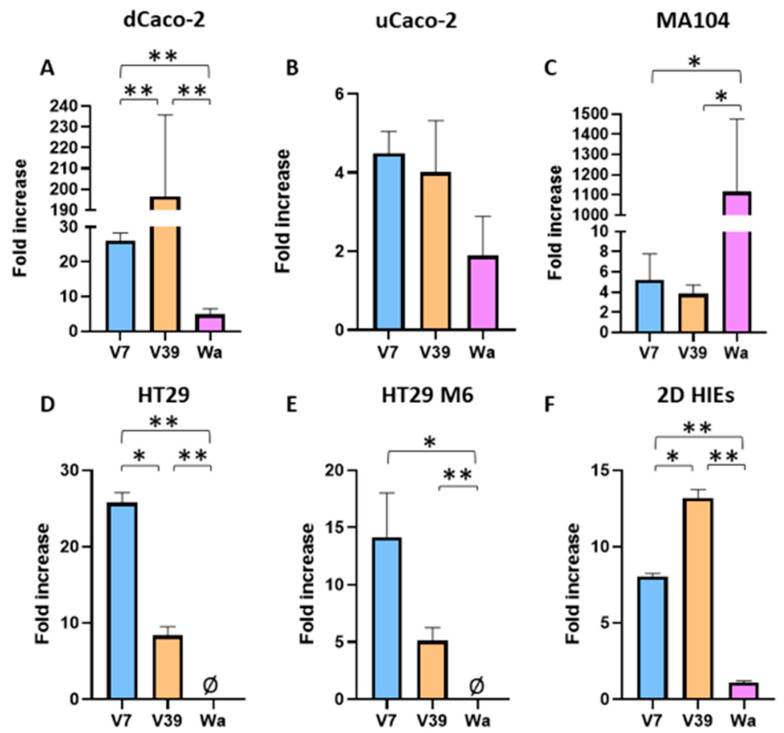
Viral replication at 72 hpi of stool filtrates V7 and V39 and the cell culture-adapted RV strain Wa in (**A**) dCaco-2 cells, (**B**) uCaco-2 cells, (**C**) MA104, (**D**) HT29, (**E**) HT29-M6, and (**F**) HIEs. *p* values < 0.05 are represented as *, *p* values < 0.01 are represented as **. Ø, no attachment; dCaco-2, differentiated Caco-2; uCaco-2, undifferentiated Caco-2; hpi, hours post infection.

Several cellular types, including dCaco-2, undifferentiated Caco-2 (uCaco-2), MA104 (monkey kidney epithelium), HT29 (human intestinal mucosecretory cells), HT29-M6 (derived from HT29 cell line with enhanced mucus secretory capacity), and monolayers of human intestinal enteroids (HIEs) were infected with human RV filtrates V7 and V39, in addition to Wa. The results showed that wild viruses replicated more efficiently in dCaco-2 cells and HIEs, whereas Wa replicated more efficiently in MA104 cells (Figure 4). RV replication after infection with the viral filtrates was lower in uCaco-2 than in dCaco-2, whereas the Wa strain replicated similarly in both cell types. In contrast to V7 and V39 RV, the Wa strain did not attach or replicate in HT29 and HT29-M6 cells. Wa attached but did not replicate in the HIE model. In contrast, V7 and V39 RV from clinical samples attached and replicated in enteroid-derived monolayers at yields similar to those of dCaco-2.

### 2.3. The VP8* Protein from V7 RV Recognizes the H1 Antigen with Better Avidity Compared to Wa

**Figure 5:**
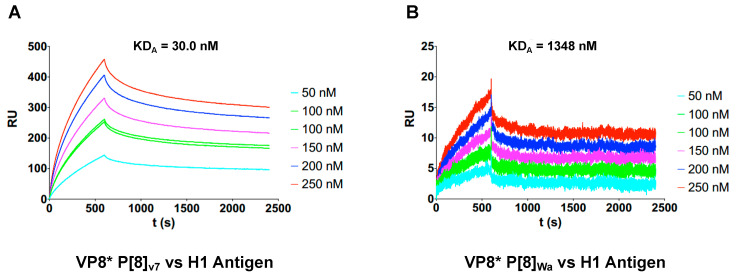
Characterization of VP8* binding to the H type-1 antigen using SPR. (**A**) shows the sensorgram of the interaction betweenVP8*_V7_ and the H type-1 antigen obtained using SPR. (**B**) shows the sensorgram of the interaction between VP8_Wa_ and the H type-1 antigen obtained using SPR.

The replication experiments suggested the role of the interaction of human RVs with HBGAs present in the gut and glycocalix of the relevant models for RV replication, including HIEs. To further explore this hypothesis, the VP8* protein from the reference strain Wa and one of the clinical samples (V7) were expressed and purified as GST-tagged proteins. The expressed proteins were utilized in SPR experiments against the H type-1 antigen. The results showed that the apparent affinity (avidity, KD_A_) of V7 VP8* was 30 nM, whereas the avidity of VP8* from the Wa strain was 1348 nM, indicating a 45-fold improvement in avidity for V7 (Figure 5), providing a possible explanation for the lack of attachment of the Wa strain to the HT29 cell lines and the poor replication in Caco-2 cells and HIEs.

## 3. Discussion

For many years, the role of the HBGAs in human RV infections was overlooked. In the last decade, several experimental studies have shown that the FUT2 polymorphisms have an implication in the susceptibility to RV infection, specifically of the P[8] genotype [34]. Since then, many reports have shown that HBGAs play an important role in human RV infections [20], especially in the severity of RV diarrhea because secretor-positive individuals are more prone to suffer from acute symptoms after RV infections [35]. In addition to the evidence obtained from infections in humans, it has been shown that the VP8* protein from human RV binds HBGAs in a genotype-dependent manner [20]. In a previous study, we determined that the different susceptibility in FUT2-negative individuals could be partially explained by the fact that VP8* from the P[8] genotype binds the H type-1 antigen with higher affinity than the non-fucosylated H type-1 precursor [24]. Despite the evidence shown in humans and at the molecular level, studies performed in vitro are discrepant because no role of HBGAs in cell cultures or HIEs was shown for RV infections [14,36].

In this scenario, we speculated that the differences observed in vivo and in vitro could be due to the adaptation of culturable human RV strains (e.g., the Wa strain, that is widely employed in RV research). Because Wa was adapted to cell culture in cell lines, such as MA104, that do not express HBGAs on their surface, the virus might have partially lost its ability to attach to HBGAs. This would have a negative impact on their propagation in in vitro systems where HBGAs could play a role in viral infectivity. To test this hypothesis, we decided to culture human RV directly from stool filtrates, similar to how other human enteric viruses (e.g., noroviruses) are routinely studied in vitro [37], and to compare them with Wa. Because Caco-2 cells are enterocytes and have been used by us and others to study the role of HBGAs in norovirus attachment experiments [38], this cell line was selected to study the replication of human RV from clinical samples. The success rate of the model was 9.75%: 4 of 41 RV-positive stool samples were able to promote RV replication. A possible explanation for this low percentage could be the presence of potential inhibitors present in the stool filtrates (i.e., bacterial metabolites with antiviral activity [20]), which would impair replication in the cell cultures, although low initial viral loads or additional factors such as the viral strain cannot be excluded.

The culture-adapted Wa strain efficiently replicated in MA104 cells compared to the rest of the cells or the in vitro model (HIE) employed here, in which the replication was limited or absent. This fact reflects a tropism in the cell line to which Wa was adapted. In addition to other differences related to its origin and tissue type, it is known that MA104 cells do not display HBGAs on their surface. It is reasonable to argue that this might be the reason why when the in vitro models that are rich in HBGAs and mucine (e.g., HT29 and HT29-M6 cells and HIEs) were challenged using Wa, viral replication was not observed under our experimental conditions. Interestingly, RV from the V7 and V39 stool filtrates (belonging to the G9P[8] genotype) were able to bind and replicate in all the HBGA-displaying models, including the monolayers of the HIEs that represent the most accurate model to study virus–host interaction in vitro. In contrast, replication of the same clinical samples in MA104 cells was low. Furthermore, an enhanced capacity to replicate was observed in dCaco-2 cells, which express a series of markers, including glycosylation. The inefficiency of Wa to replicate in Caco-2 cells was shown to be irrespective of the differentiation status of this cell model.

Moreover, we decided to compare the binding capability of VP8* from one of the clinical virus isolates (V7) and VP8* from the Wa strain. To this aim, we used the SPR approach, in which the multivalent H type-1 antigen was immobilized on the surface of a chip and VP8* was used as the analyte. In this setup, we obtained a quantitative value of KD that reflects the avidity (more than the affinity) of the interaction and allows the establishment of differences in the binding capacity of each VP8*. Our results showed that VP8* from the V7 sample had 45 times more avidity than VP8* from the Wa strain for the H type-1 antigen. This result further indicates that the adaptation process of the Wa strain to MA104 cells impaired its ability to recognize relevant HBGAs in human RV infections. However, a non-negligible fraction of the viral strains included in this study was unable to replicate in vitro, even accounting for VP4 proteins similar to successful replicating V7 and V39 RV samples, indicating that additional factors modulate in vitro infectivity in wild RV strains.

Our results indicated the relevance of using non-cell culture-adapted human RV to investigate the initial steps of RV interaction with their natural host cells. The findings of this study indicate that adapted viruses may not be appropriate tools to investigate the role of glycobiology in in vitro human RV infections.

## 4. Materials and Methods

### 4.1. Human Rotavirus Stool Filtrate Preparation

RV-positive fecal samples were obtained from the Hospital Clínico Universitario de Valencia. The G and P genotypes of the samples were studied using semi-nested PCR following the procedures of the EuroRotaNet surveillance network [39]. All the selected stool samples belonged to the P[8], G1, G9, and G12 genotypes.

Stool filtrate preparation was performed as previously described [37] with modifications. First, 0.5 g of RV-positive stool was weighed in a 15 mL conical tube and diluted in 4.5 mL of TNC 1× buffer (20 mM Tris HCl pH 8, 100 mM NaCl, 1 mM CaCl_2_). The solids were broken up using a 1 mL pipette and then vortexed for 30 s, followed by a 5 min incubation at room temperature, and then they were vortexed for 30 s. Then, the sample was sonicated 3 times for 30 s on ice (Branson Sonifier 250, Thermo Fisher Scientific, Alcobendas, Spain), with a 1 min period between each pulse (output control set to 7). Afterwards, the suspension was centrifuged at 4500× *g* for 10 min at 4 °C to remove debris. The supernatant was collected in a new 15 mL centrifuge tube and centrifuged again at 4500× *g* for 10 min at 4 °C.

The supernatant was then filtered sequentially using 5, 1.2, 0.8, 0.45, and 0.2 µm syringe filters. The resulting 10% stool filtrate was aliquoted and stored at −80 °C. The filters were purchased from Merck (Darmstadt, Germany) and Whatman (Thermo Fisher Scientific). 

### 4.2. Cell Culture Maintenance

The cell lines Caco-2 and MA104 were maintained in minimum essential medium (MEM), which was supplemented with 1% penicillin and streptomycin, 1% glutamine, 1% nonessential amino acids, and 10% fetal bovine serum (FBS). HT29 and HT29-M6 cells were grown in Dulbecco’s modified Eagle’s medium (DMEM) supplemented with 1% penicillin and streptomycin, 1% sodium bicarbonate, and 10% FBS. All cell types were grown at 37 °C under a humidified atmosphere with 5% CO_2_ in T75 flasks. Media and supplements were purchased from Gibco (Thermo Fisher Scientific).

Once the flasks achieved 80–90% confluency, the cells were rinsed with 7 mL of DPBS (Corning, Thermo Fisher Scientific) and detached using 1 mL of trypsin-versene (Lonza, Thermo Fisher Scientific) for 10 min at 37 °C. The cells were then resuspended using 9 mL of complete medium. Afterwards, the cells were counted, and the cell viability was checked using a 1:1 dilution with trypan blue solution (Merck) and counting in an automated cell counter TC20 (Biorad, Alcobendas, Spain). The cells were then transferred to 96-well plates with flat bottoms, where 10^5^ cells per well in a 200 µL volume were deposited to ensure full confluency in 24 h. In the case of Caco-2, differentiation took place after 11 days, and infection experiments were performed after 14 days. During this time, the media was changed three times. For infections in nondifferentiated cells, the experiments were performed the following day after confluence was reached.

### 4.3. Infection of Cell Lines with Human Rotavirus

Once cells were in the proper conditions, RV infection was performed. In every infection experiment, 2 or more plates were used. One of them was used as a 1 hpi timepoint to assess viral adhesion. Viral replication data were obtained by comparing the amount of virus attached to the cells (1 hpi) and the amount of virus present at the other timepoints. The experiments were always performed in triplicate.

Viral preparations were thawed at RT and diluted 1:100 in complete medium MEM or DMEM (depending on the cellular type) without FBS and supplemented with 10 µg/mL trypsin IX (Sigma) to activate the virus. The viral dilutions were incubated at 37 °C under a humidified atmosphere with 5% CO_2_ for 1 h.

In the meantime, cells were washed twice using complete MEM/DMEM without FBS. Then, 180 µL of complete medium without FBS supplemented with 1 µg/mL trypsin IX was added to each well. Once viral activation was complete, 20 µL of 1:100 viral dilutions were added to the corresponding wells (final viral dilution of 1:1000). At the dilution used for infecting cells, the viral load per well ranged from 4.4 × 10^7^ to 1.88 × 10^11^ genome equivalents (Supplementary Table S1). After 1 h of infection at 37 °C under 5% CO_2_ atmosphere, the inoculum was pipetted off and 100 µL of complete MEM/DMEM without FBS supplemented with 1 µg/mL trypsin IX was added to each well. The 1 h timepoint plate was frozen at −80 °C, and the other plates were incubated at 37 °C under 5% CO_2_ until each targeted timepoint.

The experiments performed in 96-well plates were:

- Infection of two 96-well plates with dCaco-2 (timepoints of 1 hpi and 72 hpi) to screen for viral infectivity of collected viral filtrates. Those with the ability to infect were used in the following experiments;
- Infection of five 96-well plates with dCaco-2 cells (timepoints of 1, 24, 48, 72, and 96 hpi) To determine the peak of infection for selected filtrates;
- Infection of two 96-well plates with dCaco-2 cells, uCaco-2 cells, MA104, HT29, and HT29-M6 (timepoints of 1 hpi and 72 hpi) to compare the ability of the virus to infect each cell type.

Each experiment was performed in triplicate, and the Wa strain was used in all experiments.

### 4.4. Human Intestinal Enteroid (HIE) Culture and Monolayer Preparation

Secretor-positive HIEs derived from human jejunal biopsy (J2 cell line) were provided by Prof. Mary K. Estes (Baylor College of Medicine, Houston, TX, USA). Undifferentiated 3D HIEs and differentiated monolayers were maintained and produced using commercial IntestiCult™ (INT) Organoid Medium Human media (STEMCELL Technologies Inc., Saint Égrève, France), as originally described by Ettayebi et al. [37], with the modifications included in Randazzo et al. [40].

Specifically, HIE cultures were maintained and propagated as undifferentiated 3D cultures embedded in Matrigel using proliferation INT medium (INTp), prepared by mixing equal volumes of components A and B, and supplemented with 10 mM of ROCK inhibitor Y-27632. After 7 days, highly dense 3D cultures were dissociated into a single-cell suspension and plated as undifferentiated monolayers. To this end, the domes were broken, and the cells were resuspended in Gentle Cell Dissociation Reagent (STEMCELL Technologies Inc.). After 10 min incubation on a rocking platform, dissociated cells were pelleted for 5 min at 200× *g*, resuspended in INTp, and seeded onto a 96-well plate previously coated with collagen IV. After 24 h at 37 °C in 5% CO_2_, INTp was replaced with differentiation medium (INTd) to induce monolayer differentiation. INTd was prepared by mixing an equal volume of component A and CMGF-medium (consisting of advanced DMEM–F-12 medium supplemented with 100 U/mL penicillin-streptomycin, 10 mM HEPES buffer, and 1 × GlutaMAX). HIE cell monolayers that were differentiated and 100% confluent after 5 to 7 days were for RV infections.

### 4.5. HIE Infection with RV and Stool Filtrates

Two sets of 96-well plates with 100% confluent differentiated HIE monolayers were inoculated in triplicate with 100 μL of each RV sample and incubated at 37 °C for 1 h. After the inoculum was removed, the monolayers were washed twice with CMGF-medium, and 100 μL of INTp was added to each well. For each set of infections, one 96-well plate was immediately frozen at −80 °C (1 hpi), and the second plate was incubated at 37 °C and 5% CO_2_ for 48 h (48 hpi) and then frozen. Finally, RT-qPCR was used to determine the amount of RV RNA from HIE monolayers at 1 and 48 hpi to evaluate RV replication.

### 4.6. RNA Extraction, RT-qPCR and 2^−ΔΔCt^

The plates of interest were thawed at 37 °C, and the supernatants and cells were collected in a microcentrifuge tube. Viral RNA was extracted using the Viral RNA extraction Kit (Macherey Nagel, Cultek, San Fernando de Henares, Spain). To determine whether viral replication had taken place, qPCR was performed in Exycler 96 (Bioneer, Paralab Bio, Barcelona, Spain). The master mix contained NZYSpeedy qPCR Probe Master Mix at 1× (NZY), forward and reverse primers and probe of RV at 0.25 µM, forward and reverse primers and HPRT1 probe of cells at 1× (IDT), RT SSIII (20 U/sample, Invitrogen, Thermo Fisher Scientific), and DEPC (Invitrogen, Thermo Fisher Scientific). The conditions were as follows: 50 °C 30 min; 95 °C 10 min, 45 cycles of 95 °C 15 s, 60 °C 1 min. The replication of the virus was quantified using the 2^−ΔΔCt^ method.

### 4.7. Immunofluorescence

Caco-2 cells were inoculated in 24-well plates (10^6^ cells per well in 2 mL). Each well contained a circular coverslip. Once per week, the medium was changed. When cells were differentiated, infection was performed. First, the clinical virus isolates V7 and V39 were activated at a 1/100 concentration in MEM medium without FBS supplemented with 10 µg/mL trypsin IX. After 1 h of incubation at 37 °C under 5% CO_2_, cells were washed twice with MEM medium without FBS. A total of 450 µL of medium without FBS supplemented with 1 µg/mL trypsin IX was added to each well, together with 50 µL of the activated virus (1/1000 final viral dilution). After 1 h of incubation at 37 °C and 5% CO_2_, the inoculum was removed and 1 mL of medium without FBS supplemented with 1 µg/mL trypsin IX was added. The plates were incubated for 24 h at 37 °C under a humidified atmosphere with 5% CO_2_. The medium was removed and cells were washed twice with 1 mL of PBS 1×. Fixation was performed by adding 500 µL of cold methanol and incubating at −20 °C for 20 min. The cells were then washed twice again with PBS. The blocking step was performed by adding 500 µL of PBS containing 5% BSA and incubating for 30 min at room temperature. Then, each coverslip was placed face down in a 50 µL drop of the primary antibody in parafilm. The primary antibody was α-VP6 at 1/50 concentration prepared in Dako solution. After 2 h of incubation at room temperature, the coverslips were washed 3 times with PBS. The same procedure was followed for incubation with the secondary antibody (α-mouse antibody at 1/300 dilution, mixed with DAPI at 300 nM in Dako solution). After 1 h of incubation in darkness, the coverslips were washed 3 times with PBS and placed face down on a slide with a drop of fluorescent mounting medium (Dako, Merck). The circular coverslips were then covered with bigger squared coverslips and sealed with nail polish. Preparations were observed using a confocal microscope TCS-SP8 (LEICA, L’Hospitalet de Llobregat, Spain).

### 4.8. VP8* Production and Interaction Assay

VP8* (amino acids 64-224 from the VP4 protein of RV) from the V7 isolate and the Wa strain were cloned into the pGEX-2T expression vector (Thermo Fisher Scientific). Cloning, expression, and purification of N-terminal VP8*-GST fusions were performed as previously described [24].

The affinity assays were based on SPR and performed using a Biacore T100 instrument (GE Healthcare). A total of 950 resonance units (RU) of biotinylated H type-1 antigen were immobilized in a streptavidin SA sensor chip (GE Healthcare). H type-1-PAA-biotin (0042-BP, Glyconz, Auckland, New Zeland) was diluted to a concentration of 1 mg/mL in water and captured with streptavidin present in the SA sensor chip (GE Healthcare). The immobilization process was performed by conditioning the sensor chip surface with three consecutive 1 min injections of 1 M NaCl in 50 mM NaOH before biotinylated ligands were immobilized at a flow rate of 20 μL/min. The affinity assays of VP8* polypeptides to biotinylated sugar were performed at 10 °C using 1× HBS-EP buffer (0.01 M HEPES pH 7.4, 0.15 M NaCl, 3 mM EDTA, 0.005% Surfactant P20), a flow rate of 5 μL/min, a contact time of 600 s, and a dissociation time of 1800 s. The regeneration step consisted of a wash step with 10 mM of glycine-HCl pH 2 for 20 s at the same flow rate. The kinetic assays were performed using purified VP8*-GST at different concentrations (50, 100, 150, 200, and 250 nM). Each run included at least two replicates of the 100 nM sample and three blanks without the sample. The kinetic data were obtained after the analysis of sensograms performed using the BIAevaluation 2.0 software (GE Healthcare). Kinetic association (kOn) and dissociation (kOff) rates were obtained simultaneously by fitting the data to a 1:1 Langmuir binding model. Due to the multivalent presentation of the ligand, the obtained results are a reflection of the avidity of the interaction or an apparent affinity constant (KD_A_).

### 4.9. Statistical Analysis

Graphs are represented as means ± SEM. Statistical differences were determined using the GraphPad Prism 8.0.1 software and the unpaired *t*-test to compare the means between two distinct groups. A *p* value ≤ 0.05 was considered significant.

## Data Availability

Data are contained within the article or Supplementary Material.

## Supplementary Materials

The following supporting information can be downloaded at: https://www.mdpi.com/article/10.3390/ijms242417362/s1.
