# Supplementary material for: Culture of Human Rotaviruses in Relevant Models Shows Differences in Culture-Adapted and Nonculture-Adapted Strains
Source: Int J Mol Sci. 2023 Dec 11;24(24):17362. doi: 10.3390/ijms242417362 (PMC10743750; doi:10.3390/ijms242417362)
Supplement: Supplementary file 1 [file ijms-24-17362-s001.zip › ijms-2725340-supplementary.pdf]

```
              10        20        30        40        50        60        70        80        90       100
      ....|....|....|....|....|....|....|....|....|....|....|....|....|....|....|....|....|....|....|....|
Wa    TTGGGGTCATGGAGAGATAAATGATTCGACTACAGTAGAACCAATTTTAGATGGTCCTTATCAGCCAACTACATTTACTCCACCTAATGATTATTGGATA
RV7   ...............A...........A..G.....G..........................A..T...........A.......C.............
RV39  ...............A...........A..G.....G..........................A..T...........A.......C.............

             110       120       130       140       150       160       170       180       190       200
      ....|....|....|....|....|....|....|....|....|....|....|....|....|....|....|....|....|....|....|....|
Wa    CTTATTAATTCAAATACAAATGGAGTAGTATATGAAAGTACAAATAATAGTGACTTTTGGACTGCAGTCGTTGCTATTGAACCGCACGTTAACCCAGTAG
RV7   .......CC.......................C..G.................................A.....G.A..............T.......
RV39  .......CC.......................C..G.................................A.....G.A..............T.......

             210       220       230       240       250       260       270       280       290       300
      ....|....|....|....|....|....|....|....|....|....|....|....|....|....|....|....|....|....|....|....|
Wa    ATAGACAATATACGATATTTGGTGAAAGTAAGCAATTTAATGTGAGTAACGATTCAAATAAATGGAAGTTTTTAGAAATGTTTAGAAGCAGTAGTCAAAA
RV7   ............ATG............A...A...........A..A..T......G.............................G.............
RV39  ............ATG............A...A...........A..A..T......G.............................G.............

             310       320       330       340       350       360       370       380       390       400
      ....|....|....|....|....|....|....|....|....|....|....|....|....|....|....|....|....|....|....|....|
Wa    TGAATTTTATAATAGACGTACATTAACTTCTGATACTAGATTTGTAGGAATATTGAAATATGGTGGAAGAGTATGGACATTTCATGGTGAAACACCAAGA
RV7   ...T..................C.................C.C...........A.........................................G..G
RV39  ...T..................C.................C.C...........A.........................................G..G

             410       420       430       440       450       460       470       480       490       500
      ....|....|....|....|....|....|....|....|....|....|....|....|....|....|....|....|....|....|....|....|
Wa    GCTACTACTGACAGTTCAAGTACTGCAAATTTAAATAATATATCAATTACAATTCATTCAGAATTTTACATTATTCCAAGGTCCCAAGAATCTAAATGTA
RV7   .....C.....T.......AC...........GG.CGG..............................T..........................G....
RV39  .....C.....T.......AC...........GG.CGG..............................T..........................G....

             510       520       530       540       550       560       570       580       590       600
      ....|....|....|....|....|....|....|....|....|....|....|....|....|....|....|....|....|....|....|....|
Wa    ATGAATATATTAATAATGGTCTGCCACCAATTCAAAATACTAGAAATGTAGTTCCATTGCCATTATCATCTAGATCGATACAGTATAAGAGAGCACAAGT
RV7   .............C..C.....A..T..........................A.....AT................T.......................
RV39  .............C..C.....A..T..........................A.....AT................T.......................

             610       620       630
      ....|....|....|....|....|....|....|....
Wa    TAATGAAGACATTATAGTTTCAAAAACTTCATTATGGAA
RV7   ..............C.A.......G..............
RV39  ..............C.A.......G..............
```

Figure S1. VP4 sequences of Wa strain and clinical isolates RV7 and RV39. Three aligned VP4 sequences of the clinical isolates RV7, RV39 and the cell culture-adapted Wa strain are presented. Sequence alignments were obtained with Bioedit software version 7.0.5.3.

```
              10        20        30        40        50        60        70        80        90       100
      ....|....|....|....|....|....|....|....|....|....|....|....|....|....|....|....|....|....|....|....|
Wa    ATATATTAAAATCAGTGACTCGAATAATGGACTACATTATATATAGATTTTTGTTGATTACTGTAGCATTATTTGCTTTGACAAGAGCTCAGAATTATGG
RV7   ..............T.A...A.TGCG.......TT..A..T.........C.T..AC...T...TATTGC..CAC....TGTT.A.A.A..A........
RV39  ..............T.A...A.TGCG.......TT..A..T.........C.T..AC...T...TATTGC..CAC....TGTT.A.A.A..A........

             110       120       130       140       150       160       170       180       190       200
      ....|....|....|....|....|....|....|....|....|....|....|....|....|....|....|....|....|....|....|....|
Wa    ACTTAACTTACCAATAACAGGATCAATGGACGCTGTATATACTAACTCTACTCAAGAAGAAGTGTTTCTAACTTCTACGTTATGTCTGTATTATCCAACT
RV7   .A....T.....G..C..T..C..C.....TA.A.C....G.A..T..AT.A..GC.....ACA...T.G.....A..AC....CT.A........T..A
RV39  .A....T.....G..C..T..C..C.....TA.A.C....G.A..T..AT.A..GC.....ACA...T.G.....A..AC....CT.A........T..A

             210       220       230       240       250       260       270       280       290       300
      ....|....|....|....|....|....|....|....|....|....|....|....|....|....|....|....|....|....|....|....|
Wa    GAAGCAAGTACTCAAATCAATGATGGTGACTGGAAAGACTCATTGTCGCAAATGTTTCTTACAAAGGGTTGGCCAACAGGATCTGTTTACTTTAAAGAGT
RV7   ......TCA........TGGA...ACG..A.....GA.TA.TC....C...T.A..CT.G..T..A..G........T.....A..C..T........A.
RV39  ......TCA........TGGA...ACG..A.....GA.TA.TC....C...T.A..CT.G..T..A..G........T.....A..C..T........A.

             310       320       330       340       350       360       370       380       390       400
      ....|....|....|....|....|....|....|....|....|....|....|....|....|....|....|....|....|....|....|....|
Wa    ACTCAAATATTGTTGATTTTTCTGTTGACCCACAGCTGTATTGTGACTATAATTTAGTACTTATGAAATATGACCAAAGTCTTGAATTAGATATGTCAGA
RV7   .TAATG....C.C.TCA..C..AA....T.....A..T........T......G.T.....G...........TTC..CGT.A..GC.............
RV39  .TAATG....C.C.TCA..C..AA....T.....A..T........T......G.T.....G...........TTC..CGT.A..GC.............

             410       420       430       440       450       460       470       480       490       500
      ....|....|....|....|....|....|....|....|....|....|....|....|....|....|....|....|....|....|....|....|
Wa    GTTAGCTGATTTAATATTGAATGAATGGTTATGTAACCCAATGGATGTAACATTATACTATTATCAACAATCGGGAGAATCAAATAAGTGGATATCGATG
RV7   A..............TC.A...........................A..........T........G...A.A.AT...G.G.....A............
RV39  A..............TC.A...........................A..........T........G...A.A.AT...G.G.....A............

             510       520       530       540       550       560       570       580       590       600
      ....|....|....|....|....|....|....|....|....|....|....|....|....|....|....|....|....|....|....|....|
Wa    GGATCATCATGTACCGTGAAAGTGTGTCCGCTAAATACACAAACGTTAGGGATAGGTTGTCAAACAACAAACGTAGACTCATTTGAAATGATTGCTGAGA
RV7   ...CAG..T......A.A.....A.....AT.G.....G..G..T.....A.........ATT..C.....TAC..CGA.G......GA.G.G...ACA.
RV39  ...CAG..T......A.A.....A.....AT.G.....G..G..T.....A.........ATT..C.....TAC..CGA.G......GA.G.G...ACA.

             610       620       630       640       650       660       670       680       690       700
      ....|....|....|....|....|....|....|....|....|....|....|....|....|....|....|....|....|....|....|....|
Wa    ATGAGAAATTAGCTATAGTGGATGTCGTTGATGGGATAAATCATAAAATAAATTTAACAACTACGACATGTACTATTCGAAATTGTAAGAAATTAGGTCC
RV7   G...........TA...ACA..C..T........TG.G..C......C.TG..G.G..T..A.AT..C.....A...A.G...........G.....A..
RV39  G...........TA...ACA..C..T........TG...........C.TG..G.G..T..A.AT..C.....A...A.G...........G.....A..

             710       720       730       740       750       760       770
      ....|....|....|....|....|....|....|....|....|....|....|....|....|....|....|
Wa    AAGAGAAAATGTAGCTGTAATACAAGTTGGTGGTTCTAATGTGTTAGACATAACAGCAGATCCAACAACTAATCC
RV7   ...............GA.TG.......C.....C..AG..........T..T..............T...GCA..
RV39  ...............GA.TG.......C.....C..AG..........T..T..............T...GCA..
```

Figure S2. VP7 sequences of Wa strain and clinical isolates RV7 and RV39. Three aligned VP7 sequences of the clinical isolates RV7, RV39 and the cell culture-adapted Wa strain are presented. Sequence alignments were obtained with Bioedit software version 7.0.5.3.

Table S1. Viral loads of each one of the clinical filtrates utilized to infect the different cell lines and the HIEs. The viral load indicates the number of genome equivalents calculated by qPCR as previously described (Jothikumar, et al., 2009, doi: 10.1016/j.jviromet.2008.09.025).

Strain	Viral load/well
**V1**	$2.71 \cdot 10^{9}$
**V3**	$7.62 \cdot 10^{9}$
**V5**	$3.54 \cdot 10^{10}$
**V6**	$5.58 \cdot 10^{10}$
**V7**	$1.69 \cdot 10^{10}$
**V9**	$4.3 \cdot 10^{10}$
**V10**	$6.83 \cdot 10^{10}$
**V11**	$1.2 \cdot 10^{11}$
**V12**	$2.25 \cdot 10^{10}$
**V13**	$2.26 \cdot 10^{10}$
**V14**	$1.88 \cdot 10^{11}$
**V15**	$2.6 \cdot 10^{10}$
**V16**	$1.4 \cdot 10^{10}$
**V17**	$3.43 \cdot 10^{9}$
**V18**	$7.84 \cdot 10^{8}$
**V22**	$6.62 \cdot 10^{8}$
**V28**	$4.4 \cdot 10^{7}$
**V30**	$2.04 \cdot 10^{9}$
**V31**	$3.08 \cdot 10^{9}$
**V39**	$2.1 \cdot 10^{9}$
**V41**	$2.33 \cdot 10^{9}$
